# The Present Status of Koch’s Methods

**Published:** 1891-04

**Authors:** 


### THE PRESENT STATUS OF KOCH'S METHOD.

---

We have taken some pains to ascertain from the exchanges which come to our desk, both home and foreign, the general sentiment in the profession in regard to this, our latest remedy for tuberculosis. Although sufficient time has not yet elapsed to admit of definite conclusions, and although some experimenters seem to be much disappointed at the results, yet the outlook is not discouraging. The situation is well stated by Dr. J. M. DaCosta (*Medical News*): It is premature to come to any general conclusions concerning so new a remedy. In cases of consumption in which softening has commenced the lymph is of very little use; in early cases amelioration takes place in the general condition. We cannot speak of it as a curative agent. It may be said that no case has yet been cured. In lupus and tubercular disease of the joints great good has already been accomplished. Morell Mackenzie thus states the case: I believe that Koch's fluid is an agent of the highest possible value for the detection of tubercle; a remedy of great potency for certain of the slighter manifestations of tuberculosis; a palliative for some of the distressing symptoms of the severe forms of the disease; and a deadly poison in advanced or unsuitable cases.

This much, which is all that we are warranted in saying at present, simply encourages us to hope.

